# A New Option for the Management of Dyslipidemias: Advances in Research on the Small Interfering RNA Drug Inclisiran

**DOI:** 10.31083/RCM48237

**Published:** 2026-08-24

**Authors:** Xuebing Wu, Jing Zhang

**Affiliations:** ^1^Electrocardiogram Room, Yantai Yuhuangding Hospital, 264000 Yantai, Shandong, China; ^2^Department of Cardiology, The Second Norman Bethune Hospital of Jilin University, 130000 Changchun, Jilin, China

**Keywords:** cardiovascular disease, low-density lipoprotein cholesterol, PCSK9 inhibitors, siRNA

## Abstract

Atherosclerotic cardiovascular disease (ASCVD) is associated with increased morbidity and mortality rates. Numerous studies have shown that lowering low-density lipoprotein cholesterol (LDL-C) levels can reduce the occurrence of cardiovascular disease (CVD). Current data indicate that 80% of patients with ASCVD do not achieve the LDL-C targets recommended by clinical guidelines. Nearly 15% of patients with hypercholesterolemia exhibit statin intolerance, with some experiencing severe adverse reactions such as liver function abnormalities and rhabdomyolysis. Statins require daily administration, and proprotein convertase subtilisin/kexin type 9 (PCSK9) monoclonal antibodies should be injected 12 to 26 times annually. Frequent dosing reduces patient adherence. This review comprehensively discusses inclisiran, the first lipid-lowering small interfering RNA (siRNA) drug, focusing on the associated mechanism of action, pharmacology, clinical trial data, efficacy, safety, use in special populations, and adverse effects. The objective is to provide evidence to guide the appropriate clinical use of inclisiran, a novel option for lipid-lowering therapy. Inclisiran may represent another milestone in the treatment of dyslipidemia.

## 1. Introduction

A 2018 report revealed that 26% of adults had high low-density lipoprotein cholesterol (LDL-C)levels [1]. The effective management of hypercholesterolemia is essential for preventing and mitigating the risk of cardiovascular disease (CVD). Research has demonstrated that a 1 mmol/L reduction in LDL-C levels results in 10%, 16%, 20% decreases in atherosclerotic cardiovascular disease (ASCVD) risk after one year, two years, and three years, respectively [2]. Statins are first-line drugs for lipid-lowering therapy. However, nearly 15% of patients with hypercholesterolemia exhibit varying degrees of statin intolerance, which may manifest as muscle-related symptoms, elevated liver enzymes, and, in rare severe cases, rhabdomyolysis [3]. These adverse effects may lead to dose reduction, treatment discontinuation, or poor adherence. In routine care settings, 53.1% of patients discontinue statin therapy at least once, with nearly 20% of patients discontinuing statins for at least 12 months [4]. Among patients receiving long-term statin therapy, only approximately 25% achieve their target LDL-C levels [5]. In addition to tolerability concerns, the need for repeated administration represents another important limitation of currently available lipid-lowering therapies. Statins generally require long-term daily oral administration, whereas proprotein convertase subtilisin/kexin type 9 (PCSK9) monoclonal antibodies require repeated subcutaneous injections. Depending on the specific agent and dosing regimen, PCSK9 monoclonal antibodies are typically administered every 2 weeks or once monthly, corresponding to approximately 12 to 26 injections per year [6]. The relatively frequent injection schedule may adversely affect long-term treatment adherence. Therefore, there remains a need for a novel therapeutic strategy. Inclisiran specifically inhibits the transcription of PCSK9 messenger RNA (mRNA) in hepatocytes, thereby suppressing PCSK9 synthesis and effectively lowering LDL-C levels. Compared with PCSK9 monoclonal antibodies, inclisiran, an siRNA drug, shuts down PCSK9 expression at its source. The twice-yearly dosing regimen for inclisiran reduces the inconvenience of frequent injections and enhances patient adherence to treatment.

## 2. Mechanism of Action of Inclisiran

**Fig. 1.**
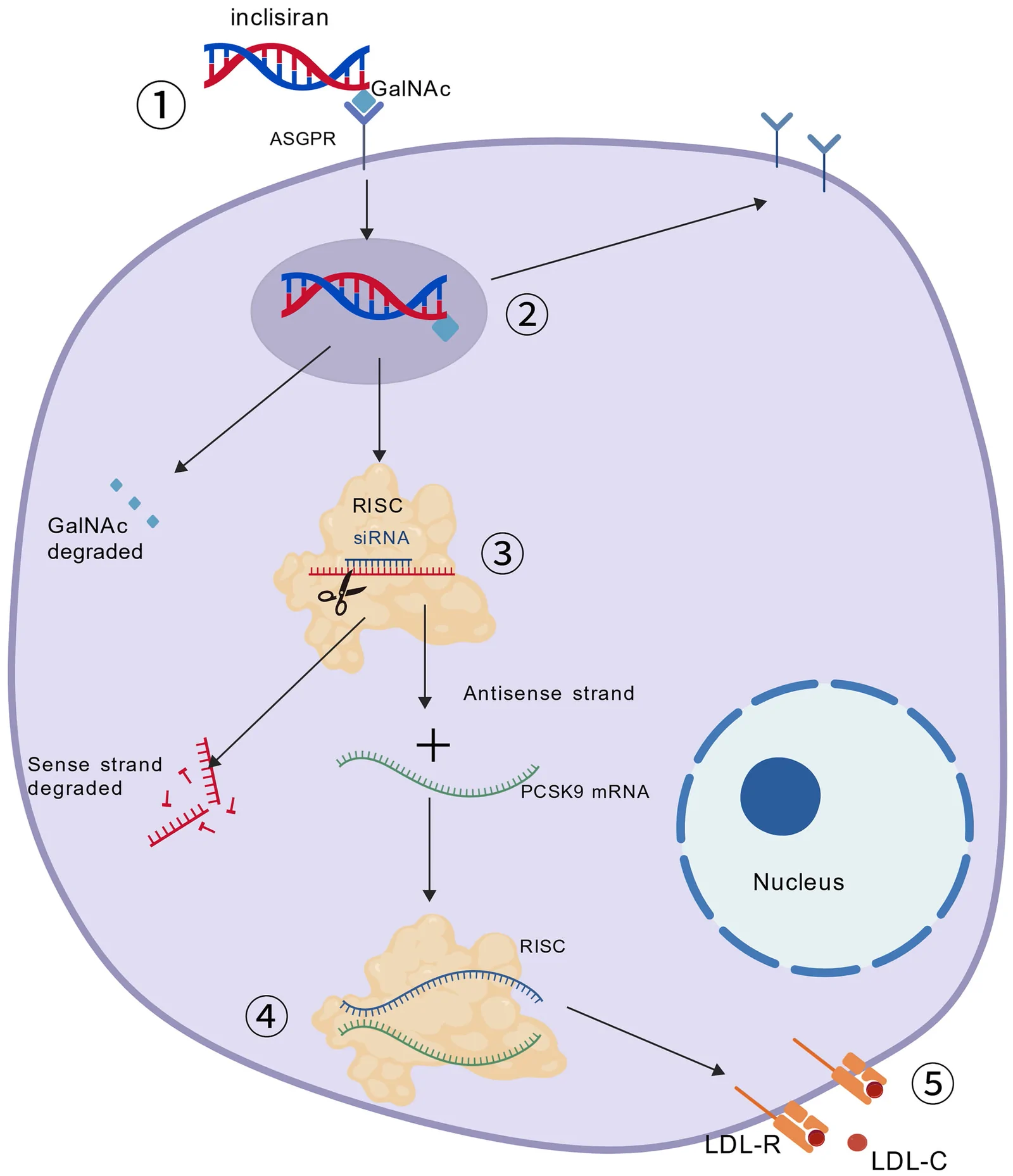
**Mechanism of action of inclisiran**. ① The N-acetylgalactosamine (GalNAc) ligand of inclisiran interacts with the asialoglycoprotein receptor (ASGPR) present on the hepatocyte surface. ② The GalNAc ligand undergoes degradation, and the double-stranded siRNA inclisiran is released into the cytoplasm, whereas ASGPR is recycled back to the cell surface. ③ Inclisiran enters the RNA-induced silencing complex (RISC), after which the sense strand is removed. ④ The amalgamation of RISC and the antisense strand subsequently attaches to proprotein convertase subtilisin/kexin type 9 (PCSK9) mRNA, leading to PCSK9 mRNA degradation and decreased PCSK9 protein synthesis. ⑤ Because less PCSK9 protein is present, more low-density lipoprotein receptor (LDLRs) are recycled to the hepatic membrane, resulting in the increased clearance of LDL-C. Created in BioGDP.

PCSK9 is a serine protease primarily produced by the liver, and is also expressed in many other tissues [7,8,9]. PCSK9 binds to the LDL-low-density lipoprotein receptor (LDLR) complex, initiating its internalization and degradation within lysosomes. This mechanism results in increased LDLR degradation, resulting in the diminished clearance of LDL particles and an increase in LDL-C levels [10]. PCSK9 can also directly affect atherosclerosis (AS) through the regulation of lipid accumulation, inflammatory factor expression, and apoptosis in the vascular wall [11,12,13]. Following subcutaneous administration, inclisiran enters the bloodstream through tissue spaces. Upon reaching the liver, the GalNAc ligand inclisiran interacts with ASGPR present on hepatocyte membrane surfaces, allowing inclisiran to enter hepatocytes via endocytosis. The GalNAc ligand undergoes degradation, and the double-stranded siRNA inclisiran is released from the endosome into the cytoplasm, whereas ASGPR is recycled back to the cell surface. The double-stranded siRNA binds to RNA-induced silencing complex (RISC), which catalyses the cleavage of PCSK9 mRNA. This reduces translational mRNA levels, decreases PCSK9 expression, decreases LDLR degradation, and ultimately decreases plasma LDL-C levels [14] (Fig. 1). PCSK9 monoclonal antibodies and inclisiran both lower LDL-C levels by reducing PCSK9 activity, but their mechanisms differ. Monoclonal antibodies bind to and block circulating PCSK9 extracellularly, while allowing the intracellular production of PCSK9 [15].

Research has indicated that inclisiran may also exert anti-AS effects through other mechanisms of action. When stimulated by oxidized LDL (ox-LDL), monocytes develop into macrophages and subsequently engulf ox-LDL. When the intake of ox-LDL surpasses the metabolic capacity of macrophages, these macrophages transform into foam cells, thereby facilitating AS progression. When RAW 264.7 cells were used as foam cell models, inclisiran notably decreased the concentrations of free cholesterol, total cholesterol (TC) and cholesterol esters in the cell culture supernatant; this prevented ox-LDL-induced lipid buildup and decreased ox-LDL uptake. The results of this experiment demonstrated that inclisiran can inhibit the conversion of macrophages into foam cells through the peroxisome proliferator-activated receptor gamma (PPARγ) pathway, thereby exerting a plaque-reversing effect in AS [16,17].

Furthermore, inclisiran can reduce endothelial apoptosis by inhibiting the maturation of inflammatory cytokines, thereby exerting an anti-AS effect. Research has indicated that NLR family pyrin domain containing 3 (NLRP3) activation leads to the recruitment of apoptosis-associated speck-like protein containing a caspase recruitment domain (CARD) (ASC) and subsequent caspase-1 activation, culminating in pyroptosis [18,19]. Endothelial cell pyroptosis leads to the release of adhesion, chemotactic, and proinflammatory factors, triggering early arterial inflammation and lipid deposition and promoting AS and plaque instability [20,21]. Inclisiran can inhibit NLRP3 activation induced by ox-LDL, thereby blocking ASC and caspase-1 activation and preventing plaque development [22].

## 3. Clinical Research Progress of Inclisiran

ORION-1 was a phase II clinical trial that assessed the efficacy of various inclisiran dose regimens in patients with increased LDL-C levels. On day 1 and day 90, participants were arbitrarily allocated to either a single-dose group (placebo, 200 mg, 300 mg, or 500 mg inclisiran) or a two-dose group (placebo, 100 mg, 200 mg, or 300 mg inclisiran). The principal endpoint was the change in the LDL-C level from baseline to day 180. In the two-dose 300 mg inclisiran group, LDL-C levels markedly decreased from baseline to day 180 in all participants; this group showed the greatest reduction in LDL-C levels, reaching 52.6% [23] (Table 1). The one-year follow-up results of the ORION-1 clinical trial [24] indicated that two 300 mg doses on day 1 and day 90 sustained a 50% reduction in LDL-C levels for up to 6 months. Therefore, a dosage schedule of 300 mg inclisiran on day 1, day 90, and subsequently biannually is an effective dosing schedule that ensures a sustained reduction in LDL-C level.

**Table 1.** **Summary of clinical trials**.

Trial	Phase	Description	Population	Key outcomes
ORION-1	Ⅱ	Assessed the efficacy of various inclisiran dose regimens	Patients at high risk for CVD who had increased LDL-C levels.	LDL-C levels reduction up to 52.6%
ORION-3	Ⅱ	Open-label extension study of ORION-1	Patients at high CVD risk	LDL-C levels decreased by 44.2% during the four years
ORION-5	III	Assessed the safety and efficacy of inclisiran in adult patients with HoFH	Adult patients with HoFH	PCSK9 levels decreased by 60%; the difference in LDL-C levels was not significant.
ORION-6	Ⅰ	Pharmacokinetic study in patients with hepatic impairment	Patients with hepatic impairment	LDL-C levels decreased by 51.9%, 53.2%, and 39.7% in the NHF group, mild HI group, and moderate HI group, without necessitating dose modification.
ORION-7	Ⅰ	Pharmacokinetic study in patients with renal impairment	Patients with renal impairment	Without the need for dose or dosing regimen adjustments
ORION-8	III	Open-label extension study of ORION-3, 9, 10, 11	Adult patients with ASCVD or risk equivalent, FH	LDL-C levels decreased by 49.4%
ORION-9	III	Assessed the efficacy of inclisiran in individuals with HeFH.	HeFH	The placebo-adjusted percentage change in LDL-C was −47.9%
ORION-10	III	Evaluated the efficacy and safety of inclisiran	ASCVD	The placebo-adjusted percentage change in LDL-C was −52.3%
ORION-11	III	Evaluated the efficacy and safety of inclisiran	ASCVD or risk equivalent	The placebo-adjusted percentage change in LDL-C was −49.9%
ORION-13	III	Evaluated the efficacy and safety of inclisiran in adolescents with HoFH	Adolescents with HoFH	The placebo-adjusted percentage change in LDL-C was −33.3%
VICTORION-Mono	III	Evaluated whether compared to ezetimibe and placebo, inclisiran monotherapy demonstrates superior efficacy in reducing LDL-C levels in a group without ASCVD.	Patients without ASCVD who had increased LDL-C levels.	The placebo-adjusted percentage change in LDL-C was −47.9%

HoFH, homozygous familial hypercholesterolemia; HeFH, heterozygous familial hypercholesterolemia; CVD, cardiovascular disease; NHF, normal hepatic function.

ORION-3 [25] which had a duration of four years, was an extension study derived from the ORION-1 study. That study sought to assess the enduring effects of inclisiran on LDL-C levels in patients at high CVD risk. Patients were divided into two groups: the inclisiran group, in which patients in the inclisiran treatment group in ORION-1 consistently received 300 mg of subcutaneous inclisiran biannually during the ORION-3 phase, and the switch group, in which patients in the placebo group in ORION-1 received subcutaneous injections of 140 mg evolocumab biweekly for the initial 360 days, and were switched to inclisiran biannually for the remaining time. The primary effectiveness endpoint was the percentage change in the LDL-C level on day 210 in the inclisiran group. The secondary endpoints were the changes in LDL-C and PCSK9 levels on day 1440 for patients in each group. In the group that received inclisiran, LDL-C levels had decreased by 47.5% on day 210 and remained reduced for more than 1440 days; during the four years, the average reduction in LDL-C levels was 44.2%, while PCSK9 levels decreased by 62.2–77.8%. In the medication-switching group, following seven injections, LDL-C levels decreased by an average of 45.3% over a three-year period, a result comparable to that observed in the inclisiran treatment group. These findings indicate that prior PCSK9 monoclonal antibody therapy did not influence the efficacy of inclisiran. The findings of that study demonstrate that biannual injections of inclisiran are well tolerated in the long term and consistently and effectively reduce PCSK9 and LDL-C levels.

ORION-10 enrolled patients with ASCVD whose LDL-C levels were ≥1.8 mmol/L. The ORION-11 trial enrolled patients with ASCVD or ASCVD equivalents (familial hypercholesterolemia (FH), type 2 diabetes or a 10-year CVD risk ≥20%) [26]. The purpose of these two phase III clinical trials was to evaluate the efficacy and safety of inclisiran in patients at high risk for CVD who have high LDL-C levels even after receiving the maximum tolerable doses of statin therapy. In both trials, participants received either 300 mg of placebo or inclisiran on day 1 and day 90, followed by biannual treatment for a total duration of 540 days. In ORION-10 and ORION-11 trials, the placebo-adjusted (intergroup differences between the inclisiran group and the placebo group) percentage changes in LDL-C levels on day 510 compared with baseline were –52.3% and –49.9%, respectively. The time-adjusted reductions in LDL-C levels from day 90 to day 540 were –53.8% and –49.2%, respectively.

ORION-8 [27] was a multicentre long-term extension study. ORION-8 evaluated whether adding inclisiran two times a year to other lipid-lowering therapies would help achieve consistent LDL-C target levels during treatment, with a follow-up duration of up to 6.8 years. When inclisiran was used, more than 70% of the patients reached the predetermined LDL-C target level, a finding that indicated that the majority of patients who received inclisiran treatment were able to consistently reach guideline-recommended LDL-C target levels. The mean percentage change in LDL-C levels was –49.4%, with no signs of attenuation in LDL-C reduction over time, demonstrating sustained and effective lowering of LDL-C levels with inclisiran. These results demonstrate that inclisiran exhibits sustained and substantial effectiveness in reducing LDL-C levels and that inclisiran is well tolerated.

VICTORION-Mono [28] was a phase III clinical trial designed to determine whether compared to ezetimibe and placebo, inclisiran monotherapy demonstrates superior efficacy in reducing LDL-C levels in a group without ASCVD. The participants were adults with no previous history of ASCVD, FH, or diabetes; with 100 mg/dL ≤ LDL-C ≤ 190 mg/dL; with a 10-year ASCVD risk prediction <7.5%; and without previous lipid-reducing therapy. Participants were randomly allocated to receive inclisiran, ezetimibe, or a placebo. The principal endpoint was the percentage change in the LDL-C level from baseline. The secondary endpoints included the absolute change in LDL-C levels and the percentage change in PCSK9 levels from baseline to day 150. The mean percentage changes in LDL-C levels from baseline to day 150 were +1.4% for the placebo group, –11.2% for the ezetimibe group, and –46.5% for the inclisiran group. The difference in LDL-C levels between the inclisiran and placebo groups was –47.9%, and that between the inclisiran and ezetimibe groups was –35.4%. The percentage change in PCSK9 from baseline to day 150 was –74.9% for the inclisiran group compared with the placebo group and –73.2% for the inclisiran group compared with the ezetimibe group. The results demonstrate that inclisiran, as a monotherapy, effectively reduces LDL-C levels and that its efficacy and safety profiles align with those observed in other phase III trials involving inclisiran in conjunction with statins. In primary prevention populations without ASCVD, compared with placebo and ezetimibe, inclisiran monotherapy showed better efficacy in lowering LDL-C levels and was well tolerated.

Heterozygous familial hypercholesterolemia (HeFH) is a hereditary disorder that affects approximately 1 in 250 individuals worldwide, with an estimated 30 million people affected. HeFH is characterized by elevated LDL-C levels from birth, which increases the risk of early-onset ASCVD [29,30]. Genetic mutations in the *LDLR* gene are responsible for more than 90% of FH cases, but mutations in other genes, including *apolipoprotein B *(*APOB*) and *PCSK9*, contribute to approximately 5% and less than 2% of cases, respectively [31]. The ORION-9 [32] aimed to assess the efficacy of inclisiran in individuals with HeFH. The principal endpoints were the percentage change in LDL-C levels from baseline to day 510 and the time-adjusted percentage change in LDL-C levels from baseline between day 90 and day 540. On day 510, the LDL-C level in the inclisiran group had decreased by 39.7%, whereas that in the placebo group had increased by 8.2%, yielding an intergroup difference of –47.9%. Between day 90 and day 540, the time-adjusted percentage decrease in LDL-C levels for the inclisiran group was –38.1%, whereas the placebo group exhibited a change of +6.2%, leading to an intergroup difference of –44.3%. LDL-C levels were markedly reduced across all genotypes in HeFH patients. Compared with the placebo, inclisiran resulted in reducing apoB, triglycerides, and TC levels but increased high-density lipoprotein cholesterol (HDL-C) levels. Lipoprotein(a) [Lp(a)] levels are an independent risk factor for ASCVD. Inclisiran can significantly reduce Lp(a) levels (by 17.2%), which may represent an additional therapeutic benefit of inclisiran treatment. Studies have shown that inclisiran effectively lowers lipid levels in adult patients with HeFH.

Homozygous familial hypercholesterolemia (HoFH) is also a hereditary condition characterized by increased LDL-C levels, potentially resulting in early-onset and progressive ASCVD. Early intervention is crucial for reducing CVD risk [33]. HoFH is a rare disease with a global prevalence of 1 in 250,000 to 1 in 360,000 people [30,34]. ORION-13 [35], a phase III clinical trial, was an inaugural study evaluating the safety and efficacy of inclisiran in adolescents with HoFH, providing key data on the application of inclisiran in paediatric populations. From baseline to day 330, the placebo-adjusted percentage change in the PCSK9 level was –60.2%, that in the LDL-C level was –33.3%, and those in the apoB, TC, and non-HDL-C levels were –23.0%, –27.8%, and –32.7%, respectively. The severity of the HoFH phenotype depends on residual LDLR activity. Patients with mutations in both alleles, resulting in the near or complete absence of LDLR activity (i.e.,* LDLR *null/null), have higher LDL-C levels and poorer clinical outcomes than do individuals with mutations in only one or two alleles, with partially reduced LDLR activity (i.e., *LDLR* null/defective or* LDLR *defective/defective) [36]. Patients with LDLR deficiency typically exhibit poor or no response to therapies that increase LDLR function. In the aforementioned study, inclisiran consistently reduced LDL-C levels regardless of genotype. The results showed that in refractory adolescents with HoFH who had lower recommended LDL-C target levels, adding inclisiran to standard therapy effectively reduced LDL-C levels while leading to favourable safety and tolerability profiles. The ORION-5 phase III trial [37] assessed the safety and efficacy of inclisiran in adult patients with HoFH. The difference in LDL-C levels between the inclisiran and placebo groups was not statistically significant. No statistically significant difference in any lipid level was detected between the two groups. The efficacy of inclisiran in adults with HoFH still requires evaluation in larger-scale, larger-population clinical trials.

Pulse wave velocity (PWV) is an independent risk factor for CVD [38]. Research has also indicated that inclisiran has favourable effects on reducing mechanical vascular injury and lowering CVD risk in patients with FH [39]. In clinical practice, PWV is the predominant metric for detecting arterial stiffness and characterizing vascular mechanical states. Higher PWV values are correlated with increased arterial damage, whereas lower PWV values reflect better vascular profiles. In a prospective observational study, after 6 months of inclisiran treatment, FH patients demonstrated significant improvements in lipid profiles and PWV. Forty-one percent of the FH patients reached target LDL-C levels, with reductions in LDL-C levels, apoB levels, non-HDL-C levels, Lp(a) levels, TC levels, and apoB/ApoA-1(apolipoprotein A1) of 41.5%, 33.7%, 35.9%, 18%, 29.5%, and 28.6%, respectively, and a 14.4% improvement in PWV. Linear regression analysis revealed a strong correlation between ΔPWV and ΔLDL-C levels (r = 0.731, *p* < 0.001). The simultaneous decrease in the LDL-C level and PWV may lead to enhanced therapeutic advantages.

The following are results of safety and tolerability analyses from seven clinical trials [40]: (1) Hepatic-related events: An increase in alanine aminotransferase (ALT) levels above three times the upper limit of normal was rare after the administration of inclisiran. After adjusting for exposure, these increases were comparable across treatment groups; (2) Muscular and kidney-related events: Elevations in creatine kinase levels (beyond five times the upper limit of normal) and creatinine levels (a 50% increase from baseline) were uncommon, with incidence rates in the inclisiran group being lower than those in the placebo group after exposure adjustment; (3) Incident diabetes and major adverse cardiovascular events(MACEs): The exposure-adjusted incidence rate (EAIR) of diabetes and MACEs in the inclisiran group was lower than that in the placebo group; (4) Anti-drug antibodies (ADAs): ADAs that were induced by inclisiran treatment were uncommon and mostly transient; they were not associated with serious adverse events that occurred after treatment or with treatment-emergent adverse events (TEAEs) that caused discontinuation of the drug; and (5) TEAEs: When inclisiran was used, the EAIR of safety events, in addition to injection site-related TEAEs, was not significantly greater than that of the placebo; TEAEs at the injection site were generally mild and transient. That study revealed that the prolonged administration of inclisiran is safe and well tolerated in various dyslipidemia patient populations treated for up to 6 years. These findings, along with the effectiveness of inclisiran in consistently lowering LDL-C levels, support its prolonged application in individuals with elevated CVD risk.

## 4. Application of Inclisiran in Special Populations

### 4.1 Individuals With Hepatic Impairment (HI)

Inclisiran is absorbed mainly by the liver via ASGPR. In cases of liver injury, ASGPR expression may be altered. When ASGPR expression or activity is reduced, plasma exposure to inclisiran may increase in patients with liver injury [41]. In the ORION-6 study [42], the exposure to inclisiran in the mild HI group (Child‒Pugh A) was 1.24 times greater than that in the normal hepatic function (NHF) group, whereas the exposure in the moderate HI group (Child‒Pugh B) was 2.03 times greater. Although plasma exposure to inclisiran increased in patients with HI, the elimination half-life (approximately 7–9 hours) and the time to reach maximum plasma concentration (Tmax) (approximately 4–6 hours) remained consistent across different groups. No inclisiran was detected in the plasma 48 hours later. On day 60, LDL-C levels decreased by 51.9%, 53.2%, and 39.7% in the NHF group, mild HI group, and moderate HI group, respectively. These results indicate that at the therapeutic dose, inclisiran is effective and safe for patients with mild and moderate HI, without necessitating dose modification. Patients with severe HI were not included in the study.

### 4.2 Individuals With Renal Impairment (RI)

Renal clearance is the primary elimination pathway for inclisiran. Plasma concentrations of inclisiran increase with increasing RI severity, leading to increased plasma exposure in patients with RI. However, inclisiran remains undetectable 48 hours after dosing and this does not influence the pharmacodynamics response to inclisiran. Specifically, the effect duration, LDL-C reduction, and PCSK9 decrease remain unaffected. Inclisiran is well tolerated, with no significant differences across various renal function groups. Inclisiran can be safely provided to patients with mild, moderate, and severe RI, without the need for dose or dosing regimen adjustments [43].

## 5. Adverse Reactions to Inclisiran

Multiple analyses [44,45,46,47] of the Food and Drug Administration (FDA) Adverse Event Reporting System (FAERS) database yielded the following results: (1) major adverse event (AE) signals: when the data were sorted by adverse drug event (ADE) signal frequency, the three most common preferred terms (PTs) were “arthralgia”, “injection site pain”, and “myalgia”. The most frequently reported system organ class (SOC) categories were “General disorders and administration site conditions”, “Musculoskeletal and connective tissue disorders”, and “Gastrointestinal disorders”. The “General disorders and administration site conditions” SOC occurred at the highest frequency; this category primarily encompasses signals such as injection site discomfort, pain, rash, blistering, reactions, discolouration, and paraesthesia. These reactions were mostly mild or transient and self-limiting. Subgroup analysis indicated that female and elderly patients were more prone to these AEs. The most frequently reported AE was injection site pain. Injection site reactions (including injection site pain, rash, pruritus, urticaria, swelling, and erythema) were localized phenomena and are common AEs associated with subcutaneous administration. These observed AEs were mild or moderate and transient and did not necessitate any intervention. Inclisiran was also linked to a higher risk of infections, such as bronchitis, gastroenteritis, nasopharyngitis, laryngitis, and influenza. The incidence of serious adverse events (SAEs) in the inclisiran treatment group was similar to or lower than that in the placebo group. The incidence of musculoskeletal and connective tissue disorder-related AEs in the inclisiran group was lower than that in the statin group, with patients generally tolerating treatment well. (2) Newly identified potential AE signals: AE signals presented in the real world that were not mentioned in drug labels or clinical trials, including hearing loss, dizziness, deafness, ear congestion, moderate insomnia, frequent urination and difficulty urinating, urinary tract infections, impaired glucose tolerance, movement disorders, macules, aphonia, pulmonary congestion, brain fog, bladder discomfort, and sinus pain. These adverse reactions have been reported in a small number of cases with low incidence rates. Clinicians should perform close monitoring, document relevant cases, and exercise caution when prescribing inclisiran, paying attention to individual variations to avoid unnecessary adverse reactions.

## 6. Future Directions

Inclisiran may be considered as an adjunctive therapy for patients who do not reach target lipid levels with statin therapy or whose treatment is restricted by adverse reactions. The combination regimen demonstrated superior safety and efficacy compared to standard statin monotherapy. A Phase III clinical trial, ORION-4, is currently underway. This study involved approximately 15,000 ASCVD patients and aimed to evaluate the effect of inclisiran on reducing MACEs in ASCVD patients. In addition to inclisiran, several siRNA therapeutics targeting different molecular pathways are currently in development and have demonstrated promising potential. These included pelacarsen, olpasiran, zerlasiran, and lepodisiran for lowering Lp(a); volanesorsen, olezarsen, and plozasiran for reducing Apolipoprotein C-III (ApoC-III); and zodasiran and solbinsiran for inhibiting Angiopoietin-like protein 3 (ANGPTL3) [48]. As research progresses, these therapies may fundamentally transform the landscape of lipid management, enhancing the quality of life for patients worldwide.

## 7. Conclusion

Inclisiran significantly reduces LDL-C and PCSK9 levels. For patients who have already received maximum tolerated doses of statin therapy but still do not achieve target lipid levels, inclisiran offers a new more effective lipid-lowering approach. Compared with other lipid-lowering medications, the biannual dosing schedule of inclisiran substantially improved patient adherence, effectively reducing CVD risk. Although the per-dose cost of inclisiran is relatively high, its long-term stable lipid-lowering effect significantly reduces the incidence of MACEs, thereby lowering subsequent treatment expenditures associated with hospitalisation, surgical interventions, and complication management. In special populations, inclisiran requires no dose adjustment and demonstrates favourable safety, thereby reducing additional healthcare costs associated with dose modifications and adverse reaction management. It offers long-term benefits from a health economics perspective. According to current relevant studies, from the perspective of national healthcare systems, the United Kingdom, the United States, and Singapore all demonstrated cost-effectiveness advantages. China announced a price reduction in January 2026, with the current price standing at RMB ¥2790.00 (USD $ 401.76) (RMB:USD = 6.94:1) per injection, exhibiting cost-effectiveness advantages following medical insurance coverage. Clinical application necessitates continuous refinement of expertise to formulate appropriate treatment plans for patients, advancing lipid management towards sustained target attainment with precision and efficiency.

## References

[1] Luo Z, Huang Z, Sun F, Guo F, Wang Y, Kao S (2023). The clinical effects of inclisiran, a first-in-class LDL-C lowering siRNA therapy, on the LDL-C levels in Chinese patients with hypercholesterolemia. Journal of Clinical Lipidology.

[2] Collins R, Reith C, Emberson J, Armitage J, Baigent C, Blackwell L (2016). Interpretation of the evidence for the efficacy and safety of statin therapy. Lancet (London, England).

[3] Fitchett DH, Hegele RA, Verma S (2015). Statin Intolerance. Circulation.

[4] Zhang H, Plutzky J, Skentzos S, Morrison F, Mar P, Shubina M (2013). Discontinuation of statins in routine care settings: a cohort study. Ann Intern Med.

[5] Gitt AK, Lautsch D, Ferrieres J, Kastelein J, Drexel H, Horack M (2016). Low-density lipoprotein cholesterol in a global cohort of 57,885 statin-treated patients. Atherosclerosis.

[6] Di Girolamo FG, Pellin L, Roni C, Biolo G, Sinagra G, Fabris E (2025). Optimizing PCSK9 inhibitor therapy: Understanding and managing suboptimal LDL-C response in clinical practice. European Journal of Internal Medicine.

[7] Ragusa R, Basta G, Neglia D, De Caterina R, Del Turco S, Caselli C (2021). PCSK9 and atherosclerosis: Looking beyond LDL regulation. European Journal of Clinical Investigation.

[8] Xia XD, Peng ZS, Gu HM, Wang M, Wang GQ, Zhang DW (2021). Regulation of PCSK9 Expression and Function: Mechanisms and Therapeutic Implications. Frontiers in Cardiovascular Medicine.

[9] Sundararaman SS, Peters LJF, Nazir S, Marquez AB, Bouma JE, Bayasgalan S (2021). PCSK9 Imperceptibly Affects Chemokine Receptor Expression In Vitro and In Vivo. International Journal of Molecular Sciences.

[10] Hess CN, Low Wang CC, Hiatt WR (2018). PCSK9 Inhibitors: Mechanisms of Action, Metabolic Effects, and Clinical Outcomes. Annual Review of Medicine.

[11] Punch E, Klein J, Diaba-Nuhoho P, Morawietz H, Garelnabi M (2022). Effects of PCSK9 Targeting: Alleviating Oxidation, Inflammation, and Atherosclerosis. Journal of the American Heart Association.

[12] Seidah NG, Garçon D (2022). Expanding Biology of PCSK9: Roles in Atherosclerosis and Beyond. Current Atherosclerosis Reports.

[13] Keeter WC, Carter NM, Nadler JL, Galkina EV (2022). The AAV-PCSK9 murine model of atherosclerosis and metabolic dysfunction. European Heart Journal Open.

[14] Migliorati JM, Jin J, Zhong XB (2022). siRNA drug Leqvio (inclisiran) to lower cholesterol. Trends in Pharmacological Sciences.

[15] Wang X, Wen D, Chen Y, Ma L, You C (2022). PCSK9 inhibitors for secondary prevention in patients with cardiovascular diseases: a bayesian network meta-analysis. Cardiovascular Diabetology.

[16] Raghavan S, Singh NK, Gali S, Mani AM, Rao GN (2018). Protein Kinase Cθ Via Activating Transcription Factor 2-Mediated CD36 Expression and Foam Cell Formation of Ly6C^hi^ Cells Contributes to Atherosclerosis. Circulation.

[17] Wang Z, Chen X, Liu J, Wang Y, Zhang S (2022). Inclisiran inhibits oxidized low-density lipoprotein-induced foam cell formation in Raw264.7 macrophages via activating the PPARγ pathway. Autoimmunity.

[18] Miao EA, Rajan JV, Aderem A (2011). Caspase-1-induced pyroptotic cell death. Immunological Reviews.

[19] Sagulenko V, Thygesen SJ, Sester DP, Idris A, Cridland JA, Vajjhala PR (2013). AIM2 and NLRP3 inflammasomes activate both apoptotic and pyroptotic death pathways via ASC. Cell Death and Differentiation.

[20] Wu X, Zhang H, Qi W, Zhang Y, Li J, Li Z (2018). Nicotine promotes atherosclerosis via ROS-NLRP3-mediated endothelial cell pyroptosis. Cell Death & Disease.

[21] Xing SS, Yang J, Li WJ, Li J, Chen L, Yang YT (2020). Salidroside Decreases Atherosclerosis Plaque Formation via Inhibiting Endothelial Cell Pyroptosis. Inflammation.

[22] Kong N, Xu Q, Cui W, Feng X, Gao H (2022). PCSK9 inhibitor inclisiran for treating atherosclerosis via regulation of endothelial cell pyroptosis. Annals of Translational Medicine.

[23] Ray KK, Landmesser U, Leiter LA, Kallend D, Dufour R, Karakas M (2017). Inclisiran in Patients at High Cardiovascular Risk with Elevated LDL Cholesterol. The New England Journal of Medicine.

[24] Ray KK, Stoekenbroek RM, Kallend D, Nishikido T, Leiter LA, Landmesser U (2019). Effect of 1 or 2 Doses of Inclisiran on Low-Density Lipoprotein Cholesterol Levels: One-Year Follow-up of the ORION-1 Randomized Clinical Trial. JAMA Cardiology.

[25] Ray KK, Troquay RPT, Visseren FLJ, Leiter LA, Scott Wright R, Vikarunnessa S (2023). Long-term efficacy and safety of inclisiran in patients with high cardiovascular risk and elevated LDL cholesterol (ORION-3): results from the 4-year open-label extension of the ORION-1 trial. The Lancet. Diabetes & Endocrinology.

[26] Ray KK, Wright RS, Kallend D, Koenig W, Leiter LA, Raal FJ (2020). Two Phase 3 Trials of Inclisiran in Patients with Elevated LDL Cholesterol. The New England Journal of Medicine.

[27] Wright RS, Raal FJ, Koenig W, Landmesser U, Leiter LA, Vikarunnessa S (2024). Inclisiran administration potently and durably lowers LDL-C over an extended-term follow-up: the ORION-8 trial. Cardiovascular Research.

[28] Taub PR, Gutierrez A, Wewers D, Garcia Cantu E, Cao H, Deck C (2025). Safety and Lipid-Lowering Efficacy of Inclisiran Monotherapy in Patients Without ASCVD: The VICTORION-Mono Randomized Clinical Trial. Journal of the American College of Cardiology.

[29] Nordestgaard BG, Chapman MJ, Humphries SE, Ginsberg HN, Masana L, Descamps OS (2013). Familial hypercholesterolaemia is underdiagnosed and undertreated in the general population: guidance for clinicians to prevent coronary heart disease: consensus statement of the European Atherosclerosis Society. European Heart Journal.

[30] Beheshti SO, Madsen CM, Varbo A, Nordestgaard BG (2020). Worldwide Prevalence of Familial Hypercholesterolemia: Meta-Analyses of 11 Million Subjects. Journal of the American College of Cardiology.

[31] Berberich AJ, Hegele RA (2019). The complex molecular genetics of familial hypercholesterolaemia. Nature Reviews. Cardiology.

[32] Raal FJ, Kallend D, Ray KK, Turner T, Koenig W, Wright RS (2020). Inclisiran for the Treatment of Heterozygous Familial Hypercholesterolemia. The New England Journal of Medicine.

[33] Cuchel M, Raal FJ, Hegele RA, Al-Rasadi K, Arca M, Averna M (2023). 2023 Update on European Atherosclerosis Society Consensus Statement on Homozygous Familial Hypercholesterolaemia: new treatments and clinical guidance. European Heart Journal.

[34] Hu P, Dharmayat KI, Stevens CAT, Sharabiani MTA, Jones RS, Watts GF (2020). Prevalence of Familial Hypercholesterolemia Among the General Population and Patients With Atherosclerotic Cardiovascular Disease: A Systematic Review and Meta-Analysis. Circulation.

[35] Wiegman A, Peterson AL, Hegele RA, Bruckert E, Schweizer A, Lesogor A (2025). Efficacy and Safety of Inclisiran in Adolescents With Genetically Confirmed Homozygous Familial Hypercholesterolemia: Results From the Double-Blind, Placebo-Controlled Part of the ORION-13 Randomized Trial. Circulation.

[36] Bourbon M, Alves AC, Alonso R, Mata N, Aguiar P, Padró T (2017). Mutational analysis and genotype-phenotype relation in familial hypercholesterolemia: The SAFEHEART registry. Atherosclerosis.

[37] Raal F, Durst R, Bi R, Talloczy Z, Maheux P, Lesogor A (2024). Efficacy, Safety, and Tolerability of Inclisiran in Patients With Homozygous Familial Hypercholesterolemia: Results From the ORION-5 Randomized Clinical Trial. Circulation.

[38] Kim HL, Kim SH (2019). Pulse Wave Velocity in Atherosclerosis. Frontiers in Cardiovascular Medicine.

[39] Bosco G, Di Giacomo Barbagallo F, Di Marco M, Scilletta S, Miano N, Esposto S (2025). Effect of inclisiran on lipid and mechanical vascular profiles in familial hypercholesterolemia subjects: results from a single lipid center real-world experience. Progress in Cardiovascular Diseases.

[40] Wright RS, Koenig W, Landmesser U, Leiter LA, Raal FJ, Schwartz GG (2023). Safety and Tolerability of Inclisiran for Treatment of Hypercholesterolemia in 7 Clinical Trials. Journal of the American College of Cardiology.

[41] Willoughby JLS, Chan A, Sehgal A, Butler JS, Nair JK, Racie T (2018). Evaluation of GalNAc-siRNA Conjugate Activity in Pre-clinical Animal Models with Reduced Asialoglycoprotein Receptor Expression. Molecular Therapy : the Journal of the American Society of Gene Therapy.

[42] Kallend D, Stoekenbroek R, He Y, Smith PF, Wijngaard P (2022). Pharmacokinetics and pharmacodynamics of inclisiran, a small interfering RNA therapy, in patients with hepatic impairment. Journal of Clinical Lipidology.

[43] Wright RS, Collins MG, Stoekenbroek RM, Robson R, Wijngaard PLJ, Landmesser U (2020). Effects of Renal Impairment on the Pharmacokinetics, Efficacy, and Safety of Inclisiran: An Analysis of the ORION-7 and ORION-1 Studies. Mayo Clinic Proceedings.

[44] Huang ZT, Zhang WH, Wang DZ, Zhang YJ, Duan YB, He XY (2026). Major adverse events associated with lipid reduction in inclisiran: a pharmacovigilance research of the FAERS database. Expert Opinion on Drug Safety.

[45] Li B, Chen Y, Zhang Y, Qian M, Shan Q, Qian J (2026). Adverse events associated with inclisiran: a real-world pharmacovigilance study of FDA adverse event reporting system (FAERS). Expert Opinion on Drug Safety.

[46] He Y, Guan X, Zhang Y, Zhu Z, Zhang Y, Feng Y (2024). Analysis of Inclisiran in the US FDA Adverse Event Reporting System (FAERS): a focus on overall patient population and sex-specific subgroups. Expert Opinion on Drug Safety.

[47] Cai X, Peng S, Mu S, Lei S, Li J, Tang X (2025). Adverse events associated with inclisiran: a real-world disproportionality analysis based on the FAERS database. Expert Opinion on Drug Safety.

[48] Kosmas CE, Bousvarou MD, Tsamoulis D, Gianniou M, Papakonstantinou EJ, Rallidis LS (2025). Novel RNA-Based Therapies in the Management of Dyslipidemias. International Journal of Molecular Sciences.

